# Genome Sequences of Chancellor, Mitti, and Wintermute, Three Subcluster K4 Phages Isolated Using *Mycobacterium smegmatis* mc^2^155

**DOI:** 10.1128/genomeA.01070-17

**Published:** 2017-11-09

**Authors:** Nicholas P. Edgington, Stephanie M. Voshell, Vassie C. Ware, Francis F. Akoto, Alexa A. Alhout, Gurvina J. Atwal, John B. Balyozian, Zachary A. Cadieux, Brianna M. Chop, Steven G. Cresawn, Netta Cudkevich, Dylan Z. Faltine-Gonzalez, Rebecca A. Garlena, Blair J. Gilmer, Lee H. Graham, Matthew S. Grapel, Maaz M. Haleem, Deborah Jacobs-Sera, Margaret A. Kenna, Maryam A. Khan, Taylor N. Klein, Jamie B. Korenberg, Brooke P. Lichak, Catherine M. Mageeney, Lauren N. McKinley, Kourtney R. Mendello, Cameron M. Myers, Alexander T. Nguyen, Bryan A. Pasqualucci, Welkin H. Pope, Lauren M. Pyfer, Wascar A. Ramirez, Julia R. Reisner, Daniel A. Russell, Paulene A. Sapao, Virginia C. Saux, Inderjeet Singh, Ty H. Stoner, Rachel H. Swope, Matthew J. Thoonkuzhy, Madeleine L. Walters, Lauren A. Vargas, Croldy A. Veliz, Kevin D. Zhang, Caitlin M. Zuilkoski, Graham F. Hatfull

**Affiliations:** a Department of Biology, Southern Connecticut State University, New Haven, Connecticut, USA; b Department of Biological Sciences, Virginia Tech, Blacksburg, Virginia, USA; c Department of Biological Sciences, Lehigh University, Bethlehem, Pennsylvania, USA; d Department of Biology, James Madison University, Harrisonburg, Virginia, USA; e Department of Biological Sciences, University of Pittsburgh, Pittsburgh, Pennsylvania, USA

## Abstract

Mycobacteriophages Chancellor, Mitti, and Wintermute infect *Mycobacterium smegmatis* mc^2^155 and are closely related to phages Cheetobro and Fionnbharth in subcluster K4. Genome sizes range from 57,697 bp to 58,046 bp. Phages are predicted to be temperate and to infect the pathogen *Mycobacterium tuberculosis*.

## GENOME ANNOUNCEMENT

Mycobacteriophages, viruses infecting mycobacterial hosts, show diversity in both genomic sequences and gene products, providing a wealth of information about phage genome evolution and insights into plausible therapeutic applications for controlling bacterial infections, such as tuberculosis (1). Indeed, phages of subclusters A2, A3, K1, and K4 have been shown to infect *Mycobacterium tuberculosis* (2). Many of these phages have been isolated by undergraduate students participating in the Science Education Alliance–Phage Hunters Advancing Genomics and Evolutionary Science (SEA-PHAGES) program (3).

Three phages were isolated by enrichment from soil samples from Blacksburg, VA (Chancellor), Bethlehem, PA (Mitti), and Ansonia, CT (Wintermute); Chancellor and Mitti were isolated at 37°C, and Wintermute at 42°C. Each produces 2- to 3-mm-diameter plaques with clear centers and turbid edges. Transmission electron microscopy revealed siphoviral morphologies. These phages are predicted to be temperate, and Mitti and Wintermute form stable lysogens that are immune to superinfection by subcluster K4 phages, and spontaneously release phage particles.

Double-stranded DNA isolated from each phage was sequenced using the Illumina MiSeq platform using 140-bp single-end reads. Chancellor and Mitti reads were assembled using Newbler, and Wintermute was assembled using SPAdes (4), each with at least 42-fold coverage. The phage genomes have 68% G+C content and lengths of 57,697 bp (Chancellor), 57,895 bp (Mitti), and 58,046 bp (Wintermute). Each genome has termini with complementary 11-base 3′ single-stranded DNA extensions (right end, 5′-CTCGCGGCCAT). Chancellor, Mitti, and Wintermute are closely related to the other four subcluster K4 members and share pairwise average nucleotide identity (ANI) values between 0.9589 and 0.9996, calculated using PyANI (https://github.com/widdowquinn/pyani). Annotations were performed using DNA Master (http://cobamide2.bio.pitt.edu/computer.htm). In each genome, 94 probable protein-encoding genes were identified with Glimmer (5) and GeneMark (6), along with one tRNA, identified using tRNAscan-SE (7) and ARAGORN (8). Functional assignments were made using HHPRED (9) and HMMER (10).

Like all subcluster K genomes, Chancellor, Mitti, and Wintermute have virion structure and assembly genes in the left half, followed by the lysis cassette that includes lysin A, lysin B, and holin genes. The immunity cassette (including integration and repressor genes) is centrally located, and the right arm includes genes involved in DNA replication. All of the genes are transcribed rightward except for the repressor (e.g., Chancellor *47*) and two genes (e.g., Chancellor *44* and *46*) flanking *int* (*45*). Gene product gp44 is similar to a family of mycobacterial lipoproteins within the antigen MPT63/MPB63 (immunoprotective extracellular protein) superfamily. Gene product gp46 is a putative membrane protein with seven transmembrane domains. Lysogenic expression of these genes could influence cellular physiology, including conferring defense against viral attack (11). Although these genomes are closely related, genes *43*, *52*, and *77* differ by small indels. Chancellor gp80 is a distant relative of other subcluster K4 homologues, showing <50% amino acid identity. By comparison, flanking genes *79* and *81* of all three phages encode proteins with >98% amino acid identity. Multiple start-associated sites (SAS) are observed in the right arm, as reported for other cluster K phages (12).

### Accession number(s).

Chancellor, Mitti, and Wintermute sequences are available at GenBank under accession numbers MF140402, KY087992, and MF140435.
